# Into the clinic: Talimogene laherparepvec (T-VEC), a first-in-class intratumoral oncolytic viral therapy

**DOI:** 10.1186/s40425-016-0158-5

**Published:** 2016-09-20

**Authors:** Hasan Rehman, Ann W. Silk, Michael P. Kane, Howard L. Kaufman

**Affiliations:** Rutgers Cancer Institute of New Jersey, 195 Little Albany Street, Room 2508B, New Brunswick, NJ 08901 USA

## Abstract

With the recent regulatory approval of Talimogene laherparepvec (T-VEC) for the treatment of advanced of melanoma in the United States, Europe and Australia, oncolytic virus immunotherapy has earned its place in the clinic. However, the adoption of T-VEC by the U.S. oncology community has been slow, and so far has been largely limited to specialized cancer centers. Limiting factors include the intratumoral route of administration, which is unfamiliar to medical oncologists, biosafety concerns related to the use of a live virus in the clinic, and the explosion of other therapeutic strategies now available for the treatment of advanced melanoma. Herein, we review the development of T-VEC, and suggest how it fits into the in the current clinical treatment paradigm, and provide pearls for drug preparation, administration, and monitoring of response to therapy.

## Introduction

There have been significant advances in the treatment of cancer over the last five years. In addition to progress in the development of targeted therapy and immunotherapy, oncolytic virus therapy has emerged as another new therapeutic option. Oncolytic viruses represent a novel class of drugs in which native or modified viral vectors are used for the treatment of cancer. The use of viruses is based on early observations in the mid-1950’s that some viruses could infect and kill leukemic peripheral blood cells in vitro. Contemporary oncolytic virus therapy mediates tumor regression through two distinct mechanisms. First, many viruses possess an innate tropism for cancer cells where they can preferentially replicate and kill established tumor cells. Secondly, the dying tumor cells can serve as a target for cross priming tumor-specific immune responses generating systemic anti-tumor immunity. This second mechanism is important since tumor cells that are not infected by virus may nonetheless be targeted for elimination by the immune system. While most oncolytic viruses are given by direct injection into established tumors, several viruses can be delivered by the intravenous route avoiding the need for tumor localization and/or complex interventional administration strategies. To date, the virus that has gained the most attention is an attenuated herpes simplex virus, type 1 (HSV-1) engineered to express human granulocyte-macrophage colony-stimulating factor (GM-CSF), termed Talimogene laherparepvec (T-VEC). This virus has been specifically adapted for selective tumor cell replication and induction of host immunity. The virus has now been tested in a prospective, randomized phase III clinical trial in which a significant improvement in durable and objective response rates were seen in patients with advanced melanoma. Based on the study results, T-VEC became the first oncolytic virus to achieve regulatory approval in the United States, Europe and Australia.

The clinical implementation of oncolytic viruses is complicated by the need to properly store, prepare and administer the virus and the use of live, replicating viruses may not be familiar to many practicing oncologists. In addition, special attention of biosafety, infection control and potential close contact transmission of the virus demand additional education and training by healthcare providers. In this brief report, we will briefly characterize the first-in-class oncoloytic virus, describe the clinical development pathway and safety profile of the vector, and discuss appropriate systems for the safe use of oncolytic viruses in the clinic. Further clinical studies of T-VEC in combination with other immunotherapy agents, as well as the emergence of multiple new oncolytic viruses, can be anticipated in the near future. The availability of T-VEC provides the treating clinician and patient with another therapeutic options for the treatment of patients with unresectable melanoma and lesions suitable for intratumoral injection.

### Construction of Talimogene laherparepvec (T-VEC)

T-VEC is a modified herpes simplex virus, type 1 (HSV-1) [1] that has undergone genetic modifications to promote selective tumor cell replication, while reducing viral pathogenicity and promoting immunogenicity. Oncolytic viruses mediate anti-tumor activity through a dual mechanism of action [2]. T-VEC replicates within neoplastic cells, and accumulation of the virions leads to lysis of the cancer cell, causing necrosis and cell death, which may release tumor-associated antigens and prime anti-tumor T cell responses. The preferential replication within tumor cells is enhanced by deletion of the herpes virus ICP34.5 genes and this also eliminated the neuropathogenicity associated with HSV-1 infection [3]. Tumor cell replication is also aided by the availability of a pool of nucleic acids in cancer cells for transcription, and the adaptation of cancer cells that often harbor disrupted anti-viral responses, specifically defects in interferon and protein kinase R (PKR) signaling. Another key modification of T-VEC is the insertion of two copies of the human cytokine granulocyte macrophage-colony stimulating factor (GM-CSF) gene. Local release of GM- CSF recruits dendritic cells and macrophages into the tumor and promotes their maturation [4]. The maturation of these cells allows the presentation of tumor antigen to T cells in the regional lymph nodes, where stimulation of tumor-specific CD8+ T cells occurs [3, 5, 6]. In development of the virus, another genetic modification, deletion of the viral ICP47 gene helps to facilitate antigen presentation and promotes anti-viral and anti-tumor immunity [3, 5]. Additional particles are released when tumor cells lyse, such as damage-associated molecular patterns (DAMPs) and pathogen associated molecular patterns (PAMPs) that also attract and stimulate inflammatory cells.

Pre-clinical data demonstrated strong lytic activity against several human tumor cell lines, including melanoma cells [5]. In the murine A20 tumor model,HSV-1 viral strains with and without GM-CSF gene expression were evaluated using a bilateral flank system wherein one flank was injected with virus and the contralateral flank was not [5]. While both HSV-1 strains resulted in regression of the directly injected tumor,, only HSV-1 encoding GM-CSF was able to inhibit tumors in the contralateral flank. Furthermore, cytotoxic T lymphocytes (CTL) recognizing the parental A20 tumor cells were only found in mice treated with HSV-1-GM-CSF. Treatment with HSV-1-GM-CSF was also associated with, protection from re-challenge with the same tumor cells, suggesting the development of long-term anti-tumor memory responses [5]. Based on these data, a modified HSV-1 with deletion of the herpes virus ICP34.5 and ICP47 genes, and encoding human GM-CSF was generated. This virus construct was termed Talimogene laherparepvec (T-VEC; Imlygic™), and was used for clinical evaluation.

### Clinical development of T-VEC

In 2006, a Phase I clinical trial of T-VEC was conducted to determine the safety profile, optimal dose and schedule, and biologic effects of therapy [7]. This study included 30 patients with refractory breast cancer (*n* = 14), head and neck cancer (*n* = 5), colorectal cancer (*n* = 2), and melanoma (*n* = 9) who had tumors in cutaneous, subcutaneous, or nodal sites that were amenable to direct injection. The trial employed a single dosing cohort and a multi-dosing arm in which an initial low dose of virus was administered to allow seroconversion of herpes virus naïve subjects. In the single-dose group, patients were exposed to a single dose of 10^6^, 10^7^, or 10^8^ plaque-forming units (PFU)/mL. In this group, the most common adverse event was pyrexia, which was mostly mild (grade 1). Local inflammation at the injected tumor site was also observed, which tended to be more severe in herpes seronegative patients, especially at higher viral doses. Injection site erythema was also commonly reported and tended to subside more quickly in seropositive patients.

In the multi-dose group, seronegative patients were given an initial dose of 10^6^ PFU /mL 3 weeks before escalation to higher viral concentrations up to 10^8^ PFU/mL, which was then repeated every two weeks [7]. Approximately one third of patients were seronegative for HSV with all seroconverting 3 to 4 weeks after the first dose. The seropositive patients also had slight increases noted in antibody titer levels [7]. To date, there has been no correlation between antibody titters and therapeutic responses or emergence of adverse events. Using a lower starting concentration of virus, the pronounced injection site reactions seen in seronegative patients were avoided even when they were subsequently administered higher viral concentrations. No dose-limiting toxicities were observed when the initial dose was 10^6^ PFU/ml. This was, thus, selected as the starting dose, and followed 3 weeks later by a higher dose of 10^8^ PFU/ml in continued two week intervals until maximum clinical response, toxicity or confirmed disease progression. This regimen, designed to reduce injection site reactions and flu-like side effects particularly in seronegative patients, became the standard dosing schedule for subsequent clinical development.

Biological activity was assessed clinically and through mechanistic correlatives including viral replication and necrosis at the injection site, GM-CSF expression, cytokine and antinuclear antibody levels [7]. No complete or partial responses occurred according to standard clinical response criteria. However, injected lesions and nearby lesions demonstrated flattening by clinical examination [7]. Histological examination of biopsies taken following injection showed inflammation and necrosis in 14 out of 19 biopsies where tumor was detected. In contrast, non-tumor cells within the tumor microenvironment showed no evidence of necrosis, thus supporting the tumor specificity of viral infection. Furthermore, areas of necrosis strongly stained for HSV proteins, while non-tumor tissue rarely stained positively for virus [7]. Baseline serostatus did not negatively impact cell necrosis following T-VEC administration.

Based on the biologic activity that was seen in the Phase I study, a Phase II trial was conducted in 50 patients with stage III and IV melanoma that was not amenable to surgery [8]. Patients received a median of six T-VEC injection cycles. In this study, 85 % patients experienced adverse effects, which were most commonly mild to moderate flu-like symptoms [8]. The overall response rate by Response Evaluation Criteria in Solid Tumors (RECIST) criteria was 26 %, with 8 patients achieving a complete response and 5 patients achieving a partial response. Notably, responses were seen in injected, uninjected skin and soft tissue sites and in visceral lesions. The responses observed were durable, as 92 % of the responses were maintained for 7 to 31 months [8]. The 1-year survival rate was 58 % in all intention-to-treat patients and 93 % in patients who demonstrated an initial objective response to T-VEC. Evidence of anti-tumor immunity was seen in a patient who achieved a complete response and was found to have increased local and systemic MART-1-specific CD8+ effector T cells following treatment [9]. The frequency of CD4 + Foxp3+ regulatory T cells and myeloid-derived suppressor cells (MDSC) was lower in tumor samples from T-VEC- treated patients compared with tumor from non-treated melanoma patients. Within the same patient, injected tumors had fewer regulatory T cells than uninjected tumors [9].

A randomized phase III study known as the OPTiM trial became the first study of an oncolytic virus to demonstrate a statistically significant clinical benefit for the treatment of melanoma [10]. In this open-label study, T-VEC was compared with recombinant GM-CSF in patients with unresectable stages IIIB, IIIC, and IV melanoma. Patients were assigned in a 2:1 randomization to receive intratumoral T-VEC (10^6^ PFU/mL followed 3 weeks later by 10^8^ PFU/mL every two weeks) or subcutaneous recombinant GM-CSF (125 μg daily for 14 days in a 28 day cycle). The primary endpoint was durable response rate (DRR), defined as a an objective response as measured by modified World Health Organization (WHO) criteria occurring within the first 12 months of therapy and lasting at least 6 months. Secondary endpoints included progression-free and overall survival, objective response rate (ORR) by independent committee assessment, and duration of response.

Of the 436 patients enrolled, 43 % had stage IV M1b/c disease and 53 % had received prior systemic therapy. Analysis of efficacy demonstrated that DRR was significantly higher in the T-VEC arm compared with the GM-CSF arm (16.3 % versus 2.1 %, p < .001). ORR was also higher in the T-VEC arm (26 %) compared with the GM-CSF arm (5.7 %). A response in 15 % of visceral metastases (all uninjected) was observed,. Median time to treatment failure (TTF) was prolonged in the T-VEC versus GM-CSF arm (8.2 versus 2.9 months). Overall median survival was 23.3 months in the T-VEC arm compared with 18.9 months in the control arm, which approached but did not cross the significance threshold (hazard ratio 0.79, *p* = .051).

T-VEC therapy in OPTiM was associated with an excellent safety profile. Similar to the experience in the earlier studies, the most common adverse events were pyrexia, chills, flu-like symptoms, injection site reactions, and fatigue. Serious treatment-related side effects were rare. The only grade 3 or 4 adverse event that occurred in more than 2 % of patients was cellulitis, which occurred in 2.1 % of T-VEC treated patients. There were no treatment-related deaths.

Based on the results of these studies, T-VEC garnered approval by the U.S. Food and Drug Administration (FDA) in October 2015 for the local treatment of unresectable lesions in patients who have recurrent melanoma after initial surgery with cutaneous, subcutaneous, or nodal lesions [11, 12]. This was followed by approval by the European Medicine Agency (EMA) with T-VEC approved for the treatment of patients with stage III unresectable or IVM1a melanoma citing the significant overall survival benefit seen in this subset of patients on analysis of the data. Regulatory approval in Australia quickly followed.

### Integration of T-VEC into melanoma treatment

The approval of T-VEC comes at a time of major transition in the treatment of metastatic melanoma. Advances in targeted BRAF and MEK in patients whose melanoma harbor mutations in BRAF and the emergence of T cell checkpoint inhibitors alone and in combination have revolutionized the therapeutic approach to advanced melanoma. While response rates are high with BRAF/MEK inhibitors, these agents are only useful in patients who have BRAF mutations and most patients will eventually develop drug resistance. The T cell checkpoint inhibitors have been associated with response rates of 11–15 % for CTLA-4 blockade and 30–40 % for PD-1 inhibitors with many patients achieving long-term benefit [13–15]. Response rates are higher with combination ipilimumab and nivolumab but treatment is often associated with significant adverse events and some patients are not able to tolerate treatment. Further, patients may present with recurrent locoregional disease in which further surgical management may not be considered curative, yet patients may not necessarily require potentially toxic systemic therapy. Thus, there are several clinical scenarios in which T-VEC may be considered.

Although improvement in overall survival in OPTiM did not cross the threshold for statistical significance, a subgroup of patients without visceral metastases did appear to derive significant benefit. In patients with stage IIIB, IIIC or IV M1a disease overall survival was significantly improved (hazard ratio 0.57, p < .001). The durable response rate in Stage IIIB/C patients was also considerably higher at 33.0 % (95 % CI, 19.1–43.9 %). An analysis of injected, uninjected non-visceral, and uninjected visceral lesions on a subset of patients from the Phase II trial illustrates the relative potency of the local and systemic effects of T-VEC [16]. Injected tumors were more likely to respond and responded more quickly compared with uninjected visceral metastases. Among lesions directly injected with T-VEC, 86/128 (67.2 %) responded (defined as a decrease in size by ≥ 30 %), including 59/128 (46.1 %), which completely resolved. Among uninjected non-visceral lesions, 60/146 (41.1 %) responded, including 44/146 (30.1 %), which completely resolved. While the response rate in injected and uninjected non-visceral lesions was very high, the response rate in visceral lesions was poor. Four out of 32 (12.5 %) responded, with only 3/32 (9.4 %) complete responses. The median time to response in injected tumors versus non-visceral uninjected tumors versus visceral uninjected tumors was 18.4 versus 23.1 versus 51.3 weeks, respectively [16]. These data suggest that T-VEC is quite effective at controlling local disease, but the systemic effects are weak and may require combination approaches.

In our clinical practice, we consider T-VEC in several situations. Patients who present with accessible lesions for injection can all be potential T-VEC candidates. In patients who have already failed other treatment regimens, elderly patients or those with significant autoimmune or other co-morbid conditions, the presence of injectable lesions leads to a discussion of T-VEC. Patients with minimal visceral disease may also be treated with T-VEC first and then proceeds with systemic therapy using T cell checkpoint inhibitors if T-VEC does not induce tumor regression. Patients with visible head and neck lesions are also good candidates for T-VEC since even localized tumor regression can lead to cosmetic improvement and reduce psychological stress.

Although no molecular biomarkers are known to improve patient selection for T-VEC therapy, the anatomic distribution of metastases is informative and predicts the chance of response [16]. Thus, T-VEC therapy can be considered for first-line therapy patients with unresectable Stage III or IV M1a disease, or in patients with relative contraindications to checkpoint inhibition, such as those with significant autoimmune disease, older age, poor performance and those who have a history of significant adverse events with other therapeutic agents.

A limiting factor to the widespread adoption of T-VEC is that not all patients have injectable disease. As melanoma has a propensity to form subcutaneous, nodal and dermal metastases, many patients will have injectable disease at some point in their treatment course. It is difficult to estimate what proportion of patients with surgically refractory disease will have tumors that are amenable to intratumoral injection in the clinic. Most clinical trials do not report this specifically, but as a surrogate, approximately 11 % of patients with unresectable disease are Stage III or Stage IV M1a [17]. This is likely an underestimate because additional patients who are categorized as Stage IV M1b/c with injectable disease.

Another consideration may be logistical barriers at the institutional or physician level to adopting new technology. Medical oncologist may be concerned that measuring and injecting tumors is more time-consuming and the biosafety precautions required may be considered too cumbersome to implement in busy clinics. A variety of medical personnel are administering T-VEC injections, including medical oncologists, surgical oncologists, dermatologists, and nurses. Administration by nurses or other healthcare professionals can lessen the hands-on time required by physicians, as is typically done with administration of IV therapies. While there is no formal training that is required, clinical sites should follow their standard operating procedures for drug handling and administration. Immunocompromised and pregnant providers should not have direct contact with storage, handling, or administering T-VEC, nor should they come into contact with the injection site [18].

There are a few contraindications to T-VEC therapy for which patients should be screened. T-VEC is contraindicated in patients who have a clinical or laboratory evidence of an active herpetic infection and in patients who require daily antiviral therapy such as acyclovir [18]. Since T-VEC is a live, attenuated virus, patients with a compromised immune status may be at risk for a life-threatening disseminated herpetic infection. Therefore, T-VEC should be avoided in severely immunocompromised patients, including those with HIV, leukemia, lymphoma, or patients on high-dose immunosuppressive therapy. There have been no studies conducted in pregnant women or children, making the use of T-VEC in this population unreasonable. Animal studies were conducted, and no effects on embryo-fetal development were noted in immunocompetent mice [18].

### Clinical administration of T-VEC

T-VEC is classified as a biosafety level 1 agent, meaning that it is not known to consistently cause disease in healthy adult humans. Many centers opt to use biosafety level 2 procedures as an added handling precaution [18]. T-VEC is prepared in a sterile biosafety cabinet by thawing frozen vials, stored at −70 °C or colder, at room temperature until conversion to liquid form. The starting concentration is 10^6^ PFU/ml (yellow-green vials) and is given initially to allow all patients to seroconvert. After three weeks, all subsequent injections utilize the higher concentration of 10^8^ PFU/ml (blue vials), which is repeated every two weeks until confirmed disease progression or unacceptable toxicity. Thawed virus can be refrigerated for 12 or 48 h depending on the concentration (10^6^ for the former, 10^8^ for the latter). However, vials should not be refrozen. [18]. Transport also requires maintaining this temperature. Caution should also be made to avoid any light exposure to the preparation during storage and thawing. When working with T-VEC, universal precautions should be followed. These basic principles include wearing protective clothing, including a gown or lab coat, gloves, and face protection [18]. If accidental exposure transpires, the area should be cleaned with water for at least 15 min. In the event of a spill, 10 % bleach solution and absorbent materials should be used to clean the area, as this has been shown to neutralize the virus after 20 min of contact [18]. A brief education of the safe handling, preparation and administration of T-VEC for healthcare providers can be very helpful.

The cutaneous, subcutaneous, and nodal lesions should be visible, palpable, or detectable by ultrasound guidance. On the day of injection, all injectable tumors should be measured clinically using calipers or a ruler and recorded on a tumor assessment worksheet, which can be annotated with a sketch of the location of the lesions on the body or photographs. It is advisable to use consistent numbering of lesions throughout the course of treatment to minimize dosing errors. The longest diameter of each lesion is used to determine the volume of T-VEC to be administered, up to a maximum of 4 ml per visit (see Table 1). In the case of multiple or large tumors, it may not be possible to inject all the tumors at each visit due to the maximum dose of 4 mL. It is generally recommended to prioritize the largest lesions first, then consider any new lesions, and then any symptomatic lesions. [18]. It is ideal to prepare one syringe for each lesion. In our center, we usually start with the largest lesion, and if this responds, we move on to smaller lesions. If a new lesion appears during treatment, this lesion is the priority. If there is a problematic lesion)i.e. painful, visible location, etc.) this lesion may be included in the injected tumors. A total of 4 ml is allowed and as many lesions as possible are injected up to 4 ml total volume at any one time. If there is a confluence of lesions, we consider the total area in the calculation of volume, and photographs can be helpful for monitoring such collections of lesions. In general, we prefer cutaneous lesions when present since these are often easier to access than nodal disease but clinically palpable or ultrasound imaged lymph nodes may also be injected.

**Table 1:** T-VEC injection volume is determined by the longest diameter of the lesion(s), up to a maximum of 4 mL at each visit

Tumor size (longest dimension)	Maximum injection volume
>5.0 cm	4.0 mL
>2.5 cm to 5.0 cm	2.0 mL
>1.5 cm to 2.5 cm	1.0 mL
>0.5 cm to 1.5 cm	0.5 mL
≤0.5 cm	0.1 mL

**Fig. 1:**
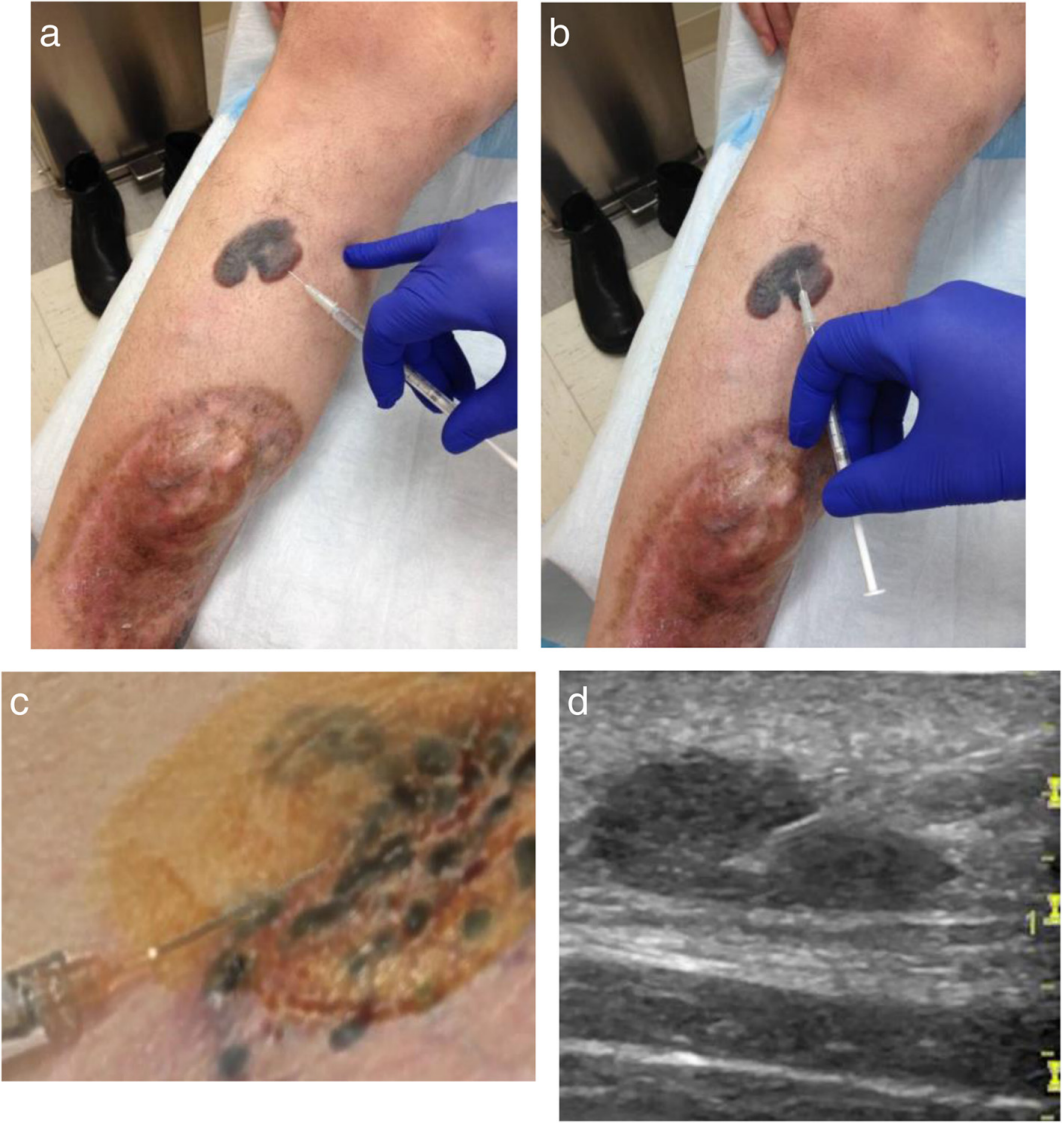
T-VEC administration. **a** Fan technique. Enter at a single insertion point as far as the needle allows within the lesion to achieve even and complete dispersion. Inject within the lesion by pulling the needle back without exiting the lesion. Redirect the needle as many times as necessary in the shape of a fan while injecting the remainder of the dose of TVEC. Continue until full administration of vial. **b** Four quadrant technique. Mark the lesion in four different quadrants 2–3 mm apart. Enter one of the marked areas with the needle and inject virus. Exit the quadrant and move on to different area and repeat in all four quadrants until complete administration of dose. **c** Clustered dermal metastases. When lesions are clustered together, they can be measured and injected as a single lesion. **d** Ultrasound. Ultrasound assistance can be used for lesions that are difficult to identify

Premedications, including local anesthetics, are not required, but may be used if a patient has previously experienced significant pain during the injections. Before injecting, the lesion in addition to the surrounding area should be cleaned with an alcohol swab and allowed to dry. T-VEC can then be injected into the lesion using the four quadrant or fan technique (see Fig. 1). A single insertion point is advised for most lesions, but multiple insertion points may be required for large, flat lesions. The needle can be slowly pulled back and redirected into multiple areas to ensure even and complete dispersion. The same preparation is required if other lesions are to be injected with the exception that a new needle is needed. If a lesion cannot be clearly palpated, ultrasound can be used to better identify the lesion and visualize the needle insertion at the time of injection.

After all lesions have been treated, pressure should be applied to the injected area for 30 s. The injected site is covered with gauze and occlusive dressing. All materials in contact with T-VEC should be discarded as outlined with universal precautions, including vials, syringes, gauze, and dressings [18]. Clinic rooms in which T-VEC was administered should be terminally cleaned with 10 % bleach-containing solution after all patients are treated. The injection site should remain covered for one week after each treatment visit. If the dressing falls off, patients should be instructed to re-dress the site. Patients should be instructed to place all soiled bandages and dressings into a sealed plastic bag, which can be disposed of in the household waste.

Patients can continue to be treated until they develop intolerance, confirmed progressive disease or complete response. Responses may be delayed or include progression of disease before regression. Median time to response in OPTiM was approximately 4 months [10]. Thus, clinical judgment is needed before stopping therapy. Qualitative responses such as flattening, softening, and eschar formation indicate biologic activity and can precede quantitative objective responses in lesion size. A biopsy may be necessary to confirm lesions that are clinically thought to have resolved completely. Residual melanophages at the site may result in persistent pigmentation in the absence of viable tumor.

### Management of adverse events

Acetaminophen or indomethacin can be used for the prevention and treatment of pain, fever or chills. Meperidine may be given for rigors following injections, although this is rarely necessary. Patients with hypophysitis or are taking low dose corticosteroids (prednisone 10 mg or less daily or equivalent) are candidates for T-VEC. These patients should be instructed that they should take extra corticosteroids if they develop a fever or flu-like symptoms to address relative adrenal insufficiency. Coordination and communication with the patient’s endocrinologist may be helpful.

To ease pain at the injection site, ice bags may be applied to the area for 5–10 min prior to injection. Oral or IV analgesics are rarely required as pre-medications. Dermal metastases tend to be the most painful due to the rich nerve endings in the dermis, while subcutaneous nodules and lymph nodes are often injected with little discomfort. Topical anesthetic such as 1 % lidocaine may be used, but care must be taken to inject that around the periphery of the tumor (not directly in the tumor) in order to prevent altered pH affecting the stability of T-VEC.

Injection site reaction, erythema, and cellulitis are common, and based on results from the OPTiM study are anticipated to occur in 27.7 %, 5.1 % and 5.8 %, respectively. Acetaminophen or indomethacin can be used for pain, erythema, and swelling. Cellulitis is a clinical diagnosis, and it is possible that local erythema and pain is due to herpes cellulitis, not superimposed bacterial infection. Herpes cellulitis from T-VEC is typically self-limiting and clears within 24–48 h whereas persistent cellulitis or associated fever and leukocytosis suggest that bacterial causes should be considered. In these cases, patients should be cultured and antibiotics started. For those with symptomatic cellulitis and other risk factors, such as diabetes mellitus, empiric treatment with antibiotics to cover for the possibility of bacterial cellulitis should be considered early.

If viremia or encephalitis is suspected, standard clinical polymerase chain reaction (PCR) testing for HSV DNA is reliable in blood and cerebrospinal fluid. These PCR probes target epitopes in the HSV-1 and HSV-2 genome that have not been modified in T-VEC, including glycoproteins, HSV DNA polymerase, or HSV thymidine kinase, which are all present and unmodified in T-VEC [19]. If a clinically significant disseminated herpetic infection occurs, treatment with acyclovir is recommended, as T-VEC is susceptible to antiviral therapy [20].

Accidental exposure is infrequent, but remains a concern. Four incidents of accidental exposure in healthcare workers have been reported to date out of over 4,000 treatment visits [21]. Patients and healthcare workers should continuously monitor themselves for clinical signs of a herpetic outbreak. Isolated incidents have occurred. Viral genomic testing determined that the herpetic infection was related to the wild-type strain, not the genetically modified product. These incidents should be reported if they occur in the patient or any contacts, including healthcare workers. Acyclovir or other anti-viral medication is indicated in the event of accidental exposure to T-VEC.

### Future directions

As T-VEC has a tolerable safety profile, is highly effective in injected lesions and promotes anti-tumor immunity, it is tempting to combine it with complementary systemic approaches to promote systemic immunity and treatment of distant, uninjected lesions. T-VEC may support immunotherapy by enhanced by IFNγ and TNFα production, which can increase PD-L1 expression, promote influx of effector CD8+ T cell to the tumor microenvironment and decrease the number of CD4 + FoxP3+ regulatory T cells [22]. Trials are underway exploring combinations with other immunotherapies, such as ipilimumab and pembrolizumab [23, 24]. In a phase I clinical trial of T-VEC and ipilimumab there were no unexpected adverse events reported and an objective response rate of 50 % was seen with 44 % of the patients experiencing durable responses lasting 6 months or greater [24]. T-VEC is also being studied in combination with chemoradiation therapy. A phase I/II dose-finding study of T-VEC in locally advanced squamous cell cancer of the head and neck (SCCHN) was conducted to define recommended dosage and interactions with chemoradiation [25]. Patients received chemoradiation therapy plus T-VEC every three weeks for a total of four doses. There were no delays to chemoradiation therapy or dose-limiting toxicities. At a median follow-up of 29 months, locoregional control was 100 %, disease-specific survival was 82 %, and relapse-free survival was 76 % [25].

Other important future studies with T-VEC will include evaluation of T-VEC injections directly into visceral tumors and neoadjuvant administration to prevent tumor recurrence following surgical resection. In addition, T-VEC will be evaluated in other tumors with studies planned in non-melanoma skin cancers, soft tissue sarcomas, rectal cancer, pancreatic cancer and others. Additional investigations into the mechanisms of anti-tumor activity, induction of host anti-tumor immunity and identification of predictive biomarkers will also be of major importance.

## Conclusions

T-VEC represents the first oncolytic virus, a new class of drugs that mediate anti-tumor activity by directly killing tumor cells and secondarily inducing systemic tumor-specific immunity. T-VEC is now approved for the treatment of melanoma after demonstrating clinical benefit in a randomized phase III clinical trial and is associated with a tolerable safety profile, consisting of mild constitutional symptoms and local injection site reactions. The integration of T-VEC into the clinic is associated with the need for healthcare provider education, universal precautions, special storage and handling procedures and comfort with local injections. T-VEC can be considered in patients with accessible lesions for injection by clinical palpation or ultrasound guided injection and is especially useful for older patients, those with contraindications to other melanoma therapy and those that need rapid locoregional control and are not surgical candidates. Further studies of T-VEC combinations, most notably with T cell checkpoint inhibitors, will be especially interesting. T-VEC is another immunotherapeutic strategy available for the treatment of advanced melanoma.

## Abbreviations

BRAF, serine/threonine-protein kinase B-Raf; CD, cluster of differentiation; CTL, cytotoxic T lymphocyte; CTLA-4, cytotoxic T lymphocyte antigen 4; DAMPS, danger-associated molecular patterns; DNA, deoxyribonucleic acid; EMA, European Medicines Agency; FoxP3, forkhead box P3; GM-CSF, granulocyte-macrophage colony stimulating factor; HIV, human immunodeficiency virus; HSV-1, herpes simplex virus, type 1; ICP, infected cell protein; INF, interferon; MDSC, myeloid-derived suppressor cell; MEK, mitogen-activated protein kinase kinase; ml, milliliters; OPTiM, Oncovex^GM-CSF^ Pivotal Trial in Melanoma; PAMPS, pathogen-associated molecular patterns; PCR, polymerase chain reaction; PD-1, programmed cell death 1; PD-L1, programmed cell death ligand 1; PFU, plaque-forming unit; PKR, protein kinase R; RECIST, Response Evaluation Criteria for Solid Tumors; SCCHN, squamous cell carcinoma of the head and neck; TNF, tumor necrosis factor; T-VEC, Talimogene laherparepvec; U.S., United States.
